# Climatic Factors and Influenza Transmission, Spain, 2010–2015

**DOI:** 10.3390/ijerph14121469

**Published:** 2017-11-28

**Authors:** Diana Gomez-Barroso, Inmaculada León-Gómez, Concepción Delgado-Sanz, Amparo Larrauri

**Affiliations:** 1 National Centre for Epidemiology, Carlos III Institute of Health, Monforte de Lemos 5, 28029 Madrid, Spain; ileon@isciii.es (I.L.-G.); cdelgados@isciii.es (C.D.-S.); alarrauri@isciii.es (A.L.); 2 Consortium for Biomedical Research in Epidemiology and Public Health (CIBER Epidemiología y Salud Pública—CIBERESP), Monforte de Lemos 5, 28029 Madrid, Spain

**Keywords:** influenza transmission, meteorological parameters, absolute humidity, rainfall, spatio-temporal mixed models

## Abstract

The spatio-temporal distribution of influenza is linked to variations in meteorological factors, like temperature, absolute humidity, or the amount of rainfall. The aim of this study was to analyse the association between influenza activity, and meteorological variables in Spain, across five influenza seasons: 2010–2011 through to 2014–2015 using generalized linear negative binomial mixed models that we calculated the weekly influenza proxies, defined as the weekly influenza-like illness rates, multiplied by the weekly proportion of respiratory specimens that tested positive for influenza. The results showed an association between influenza transmission and dew point and cumulative precipitation. In increase in the dew point temperature of 5 degrees produces a 7% decrease in the Weekly Influenza Proxy (RR 0.928, IC: 0.891–0.966), and while an increase of 10 mm in weekly rainfall equates to a 17% increase in the Weekly Influenza Proxy (RR 1.172, IC: 1.097–1.251). Influenza transmission in Spain is influenced by variations in meteorological variables as temperature, absolute humidity, or the amount of rainfall.

## 1. Introduction

The spatio-temporal distribution of several infectious diseases is linked to variations in meteorological factors, like temperature, absolute humidity, or the amount of rainfall. Influenza epidemics in temperate regions show a characteristic seasonal pattern with peak incidence occurring in winter [1]. Different studies in different countries show that in those periods when absolute humidity and the temperatures in a region are lower, the risk of influenza transmission increases [1,2,3,4,5,6,7,8,9,10,11].

According to the definitions provided by the U.S. National Oceanic and Atmospheric Administration, absolute humidity is the density of water vapour, defined as the ratio of the mass of water vapor to the volume of associated moist air, and is generally expressed in grams per cubic meter. Dew point is the temperature to which the air must be cooled for water vapour to condense and form fog or clouds. The dew point reflects the “absolute humidity” of the air. In the months in which the temperature is lower (winter), absolute humidity is lower, and, consequently, the dew point decreases [12].

The Spanish Influenza Sentinel Surveillance System (SISSS) was implemented more than a decade ago, in accordance with the established guidelines for these types of systems [13,14]. The fundamental features of the SISSS are such that it allows for the combined collection of virological and epidemiological data on influenza, and so helps to ensure early detection and characterization of circulating viruses and also assessment of their capacity for propagation within in the population [13]. This surveillance system provides weekly information on the influenza activity in Spain at different geographical levels, meaning an opportunity to study the relations between flu transmission and climatic factors in our country.

According to the classification provided by a Iberian Climatic Atlas [15], there are two large climatic zones in Spain: (a) Type B (zones with a dry climate), which is sub-divided into areas of both hot and cold desert, as well as hot and cold steppes; and, (b) Type C (temperate areas), which, according to sub-division, includes temperate zones without a dry season, but with hot or temperate summers, and areas with dry hot summers or dry temperate summers. There are also small zones of mountainous heights with cold climates. These conditions are typically in temperate climates.

There are no studies at a national level in Spain that analyze this aspect. For this reason, we aimed in this study to analyze the association between influenza activity, obtained from the SISSS, and meteorological variables in Spain, across five influenza seasons: 2010–2011 through to 2014–2015. The results could provide important information to guide the implementation of control measures for the disease during winter influenza epidemics.

## 2. Material and Methods

### 2.1. Setting

According to the Spanish Statistical Office (Instituto Nacional de Estadística, INE, Madrid, Spain), Spain had 8116 municipalities in 2014. These municipalities are aggregated into 52 provinces, which, in turn, are aggregated in to 17 autonomous regions and two autonomous cities. The classification of Köppen [15] defines various kinds of climates based on monthly averages for precipitation and temperature. It establishes temperature and precipitation ranges to distinguish between the various climates, based principally on the distribution of vegetation and the influence on human activity.

### 2.2. Influenza Activity Data

Information regarding the declared cases of influenza was obtained from the Spanish Influenza Sentinel Surveillance System (SISSS), which is comprised of 17 regional influenza sentinel networks in 17 out 19 Spanish regions, as well as influenza laboratories in every region. Briefly, in this system, Sentinel Physicians (SPs) collect information on each patient who presents with an influenza-like illness (ILI), which meets the EU ILI case definition. In addition, SPs systematically swabbed the first two patients who visited their consultation rooms each week for ILI, and subsequently sent respiratory samples for laboratory virological confirmation. As a consequence, the percentage of positive specimens of influenza virus is available weekly (weekly influenza detection rate). Epidemiological and virological data were entered weekly, and are shown on the website of the Spanish Influenza Surveillance System (http://vgripe.isciii.es/gripe).

ILI cases are aggregated at a municipal level according to the location of the weather station. Weekly ILI rates were calculated using as denominator the weekly population being monitored by each SP. For each municipality, we calculated the weekly influenza proxies, which are defined as the weekly ILI rates, multiplied by the weekly proportion of respiratory specimens that tested positive for influenza [16].

We analysed the influenza seasons from 2010–2011 to 2014–2015. For each season, eleven weeks of influenza epidemic were included in the analysis, the week of the peak and the five preceding and following weeks.

### 2.3. Meteorological Data

Daily meteorological data from 73 weather stations in Spain was obtained from the National Climatic Data Center [17]. Daily data for dew point temperature, the quantity of rainfall, and overall temperature were collected, although the latter was not incorporated into the final models. Only those weather stations that were missing no more than 5% of their data were included. In the case of missing data, the missing values were calculated using moving averages. Weekly averages for mean daily temperature, daily dew point, and weekly accumulated rainfall were obtained.

“Meteorological areas” were calculated using the coordinates from the weather stations. The weather station that was closest to the center of each municipality was assigned to the centroid of that area, with the effect that all those municipalities that correspond to the same weather station form part of the same meteorological area.

### 2.4. Statistical Analysis

The Pearson’s correlation coefficient between temperature and dew point is 0.80. To avoid the collinearity between both of the variables, only dew point was used in the final models. Three generalized linear negative binomial mixed models fitted by maximum likelihood were used to analyse the relationship between the weekly influenza proxy and the meteorological factors: dew point and quantity of rainfall. 

Model 1 only considers dew point, Model 2 only considers only weekly rainfall, and Model 3 considers both meteorological variables.

- Model 1
  $$log\tau_{ijk}=\beta_{0}+\beta_{1}sen_{11ijk}+\beta_{2}cos_{11}ijk+\beta_{3}trend\;ijk+\beta_{4}dew\;point\;ijk+area_{\;jk}+season_{k}$$
- Model 2
  $$log\tau_{ijk}=\beta_{0}+\beta_{1}sen_{11ijk}+\beta_{2}cos_{11ijk}+\beta_{3}trend\;ijk+\beta_{4}rainfall\;ijk+area_{jk}+season_{k}$$
- Model 3
  $$log\tau_{ijk}=\beta_{0}+\beta_{1}sen_{11}ijk+\beta_{2}cos_{11ijk}+\beta_{3}trend\;ijk+\beta_{4}dew\;point\;ijk+\beta_{5}rainfall\;ijk+area_{jk}+season_{k}$$

where *i*: is the *i* element in the *j* season in the *k* area, *j*: is the *j* season in the *k* area, and *k*: is the *k* area. The three models all include two random effects: a spatial effect “area”, which is the “meteorological area” and another effect, which is the “influenza season”. In addition, as fixed effects, we have included seasonality ${“\mathrm{sen}}_{11,}\cos_{11}”$, the trend for controlling the effect of the epidemic wave, and the seasonality of influenza and the meteorological variables.

For all of the predictors and associations between variables, differences were considered significant at *p* value < 0.05.

In a preliminary analysis, we did not find significant differences in influenza transmission between the different climatics zones (data not shown).

## 3. Results

### 3.1. Influenza Activity

The number of SPs participating in the five analysed influenza seasons ranged from 788 to 873, covering a percentage of the surveilled population between 2.17 and 2.44 (Table 1). While the 2011–2012 and 2012–2013 seasons had a late peak in February, the rest of the epidemics peaked during January. Higher cumulative ILI rates were observed in the A(H3N2) predominant influenza seasons 2011–2012 and 2014–2015. The proportion of influenza positivity over the seasons ranged from 45% in the 2010–2011 season to 54.5% in the 2014–2015 influenza season. The A(H1N1)pdm09 strain predominated in the 2010–2011 season, and co-circulated with A(H3N2) in the 2013–2014 season. A(H3N2) was predominant in 2011–2012 and 2014–2015, while influenza B predominated in the 2012–2013 season (Table 1).

**Figure 1:**
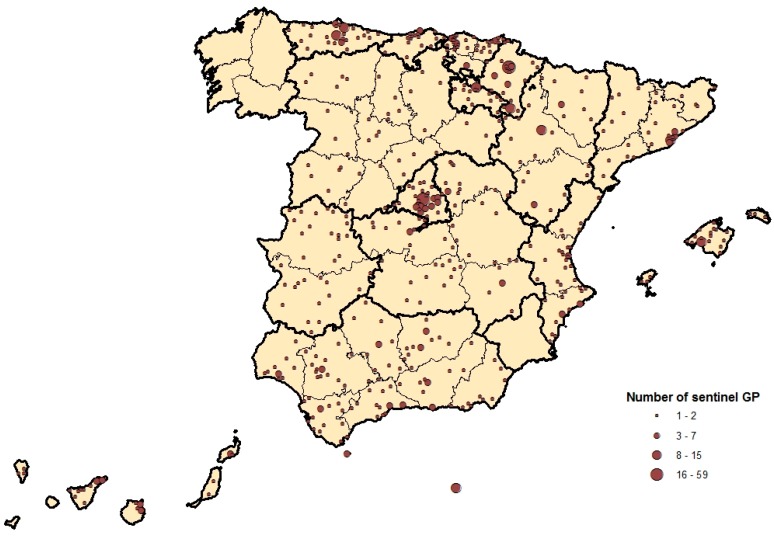
Distribution of sentinel physicians in the 2012/2013 influenza season.

Figure 1 shows the spatial distribution of the Sentinel Practitioners (SPs) across the country. The SPs cover all of the territory under surveillance; however, in large municipalities and provincial capitals there was a higher concentration of SPs than in smaller municipalities.

### 3.2. Meteorological Variables

**Figure 2:**
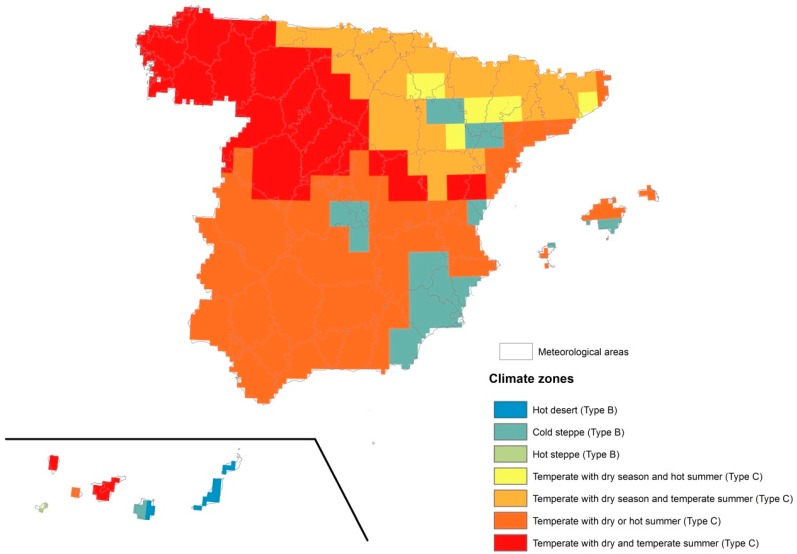
Distribution of, location of the weather stations, and the climatic areas based on Köppen’s classification.

We analysed data from 73 weather stations, with complete information and distributed across the whole territory in question, and from which 73 meteorological areas in seven climatic areas were constructed—following Köppen’s climatic classification (Figure 2).

The climatic variables in Spain during the eleven weeks of the analysed period are shown in Table 2. The weekly cumulative rainfall by meteorological region ranged from 0 to 553.21 mm. The weekly dew point varies from −13.59 to 23.46 °C. The weekly mean temperature in Spain is estimated at 16.07 °C (Table 2). 

### 3.3. Climate Factors and Influenza Activity

**Figure 3:**
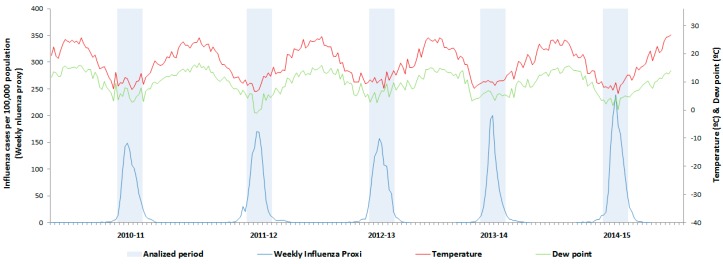
Weekly influenza proxy, mean temperature and dew point, Spain, influenza seasons 2010/2011 to 2014/2015.

Figure 3 shows the evolution of the weekly influenza proxy across the five influenza seasons that were analysed, in addition to the average weekly temperature and the average weekly dew point for the same period. The maximum values for the weekly influenza proxy coincide with the minimum temperature values for average temperature and dew point in all of the influenza seasons that were analysed.

• Model 1: Weekly influenza proxy and dew point.

An increase of 1, 5, or 10 degrees in the dew point produces a statistically significant decrease in the weekly influenza proxy (Table 3). When the dew point temperature increases by 5 degrees, the influenza proxy drops by 5% (RR: 0.954; 95% CI: 0.918–0.992).

• Model 2: Weekly influenza proxy and rainfall volume.

An increase of 1, 10, or 50 mm in the volume of precipitation produces a statistically significant increase in the weekly influenza proxy (Table 3). If the volume of rainfall increases by 10 mm over a week, then the weekly influenza proxy increases by 1% (RR: 1.010; 95% CI: 1.001–1.020) (Table 3).

• Model 3: Weekly influenza proxy, dew point and rainfall volume.

**Figure 4:**
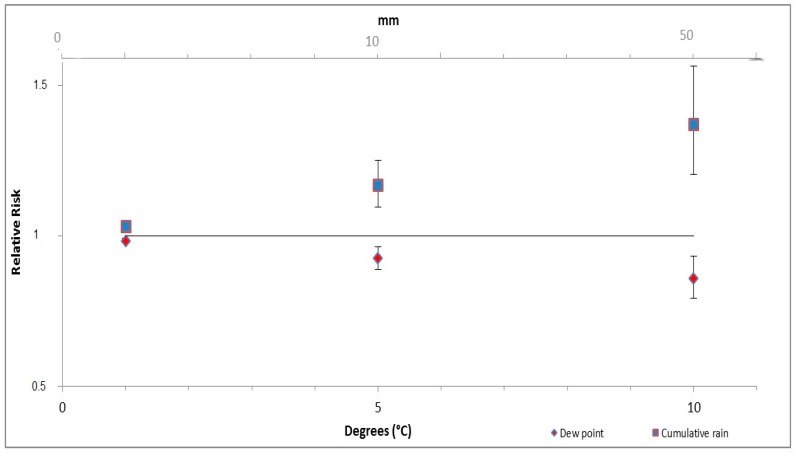
Result of the multivariate model. RR of Weekly Influenza Proxy were calculated at levels 1, 5 and 10 °C of dew point and at levels 1, 10 and 50 mm of cumulative rainfall.

The multivariate model shows a statistically significant association between the influenza activity and all three of the meteorological variables that were analysed (Table 3 and Figure 4). An increase in the dew point temperature of 5 degrees produces a 7% decrease in the Weekly Influenza Proxy (RR 0.928; 95% CI: 0.891–0.966), while an increase of 10 mm in weekly rainfall equates to a 17% increase in the Weekly Influenza Proxy (RR 1.172; 95% CI: 1.097–1.251).

## 4. Discussion 

This is the first study that was carried out at a national level that provides information on the relationship between the transmission of influenza and meteorological variables, like dew point temperature (or absolute humidity) and the quantity of rainfall. This study analyses these variables across five influenza seasons in Spain. Our results show that the transmission of influenza increases when the volume of precipitation increases, and when absolute humidity decreases.

This study shows that a decrease in absolute humidity is associated with the transmission of the influenza virus in temperate regions. The results are consistent with those that were obtained by studies carried out in other countries. While our study sets out a 2% variation in influenza incidence in relation to an increase of 1 degree in the dew point, a study carried out in The Netherlands [1] concluded that 3% of influenza transmission could be explained by absolute humidity. In addition, studies in the USA observed that a decrease in absolute humidity is associated with influenza virus transmission [6,7,18]. A study recently that was published by Soebiyanto et al. [19] studied meteorological parameters in five European regions, among which, one was Spanish, Castilla y León. They found that influenza activity is inversely proportional to absolute humidity, in line with the findings of this study. Soebiyanto et al., however, did not find any conclusive results in relation to the volume of rainfall, in contrast to our results that show a statistically significant increase in influenza transmission in relation to an increase in the quantity of precipitation. Another study carried out in Spain by Fdez-Arroyabe concluded the existence of clear interactions between climatic factors and influenza epidemics. In this work, the author highlighted the enormous value to the inclusion of meteorological in the statistical models that are used to forecast the outbreaks and temporal evolution of influenza epidemic [20].

The meteorological areas constructed represent all of the climatic zones described by Köppen in Spain [15]. In our study, we did not find significant differences in influenza transmission between the different climatic zones. This could be due to the use of the peak of the epidemic wave and the five weeks before and after at a national level, while differences could exist on the start of the epidemic between different regions that were not taken into account.

The correlation between temperature and dew point is very high at 0.8, which is expected from a meteorological standpoint. This indicates that using temperature in place of absolute humidity would have produced similar results.

The weekly influenza proxy combines epidemiological and virological surveillance data, so it can be considered as one of the best relative measure of influenza incidence that can be calculated from our surveillance system [21]. The period of analysis was restricted to the peak weeks of influenza incidence and the five weeks preceding and following, in each season, to avoid periods with low circulation of influenza viruses.

One of the limitations of this study is the spatial distribution of the weather stations. As a consequence of having only 73 weather stations distributed across the whole geographical area, it can be inferred that the meteorological parameters from the closest station that are assigned to each municipal area are not exact, as the closest weather station could be a number of kilometres distant. Furthermore, the terrain was not taken into account in the calculation of the meteorological data for a municipality. In addition, the use of weekly averages for meteorological parameters could also have affected the level of precision. Nevertheless, the weather stations are distributed across the whole of the peninsula and represent all of the principal types of climatic area, as defined by Köppen in Spain [15].

## 5. Conclusions

Influenza transmission in Spain is influenced by variations in meteorological variables as temperature, absolute humidity or the amount of rainfall. In the annual epidemics that occur during winter in Spain, the transmission of influenza increases during periods of high precipitation and in those that produce a decrease in absolute humidity. Prior knowledge of these phenomena can positively influence the adoption of adequate measures to control the disease and aid in the prioritization of health resources at a regional and national level. One possible approach would be to integrate the national and regional weather forecasting models into the seasonal and pandemic influenza surveillance by the SISSS in Spain.

## Figures and Tables

**Table 1:** Characteristics of the influenza seasons from 2010/2011 to 2014/2015.

Influenza Season	SPs (N)	Surveilled Population	Percentage of Surveilled Population (%) *	Week of Influenza Epidemic Peak	Cumulative ILI Rate (ILI Cases/100.000)	Respiratory Swabs Analysed	Positivity (%) **	Dominant Influenza Virus
2010–2011	839	1,079,753	2.38	2	2099.78	5482	45.00	A(H1N1)pdm09
2011–2012	848	1,086,983	2.37	7	2232.49	5858	50.05	A(H3N2)
2012–2013	831	1,026,896	2.22	8	2181.79	5173	51.46	B
2013–2014	873	1,137,848	2.44	4	1906.30	5060	50.30	A(H1N1)pdm09/A(H3N2)
2014–2015	788	1,006,183	2.17	5	2477.57	5101	54.48	A(H3N2)

** Proportion of respiratory specimens positive for influenza; * Percentage of population under surveillance with respect to the total population of the autonomous community; ILI: influenza-like illness.

**Table 2:** Descriptive statistics for climate variables in Spain 2010/2011 to 2014/2015.

Climate Parameters	Min	Max	Mean	SD
Weekly cumulative rainfall (mm)	0	553.21	9.89	20.89
Weekly dew Point (°C)	−13.59	23.46	8.9	5.43
Weekly temperature (°C)	−8.63	33.01	16.07	6.86

**Table 3:** Weekly influenza and weekly mean dew point and cumulative precipitations proxy. Results of the univariate (models 1 and 2) and multivariate model (model 3).

	Univariate (Models 1 and 2)		Multivariate (Model 3)	
*Dew point (°C)*				
	RR *	(95% CI)	RR *	(95% CI)
1	0.991	(0.983; 0.998)	0.985	(0.977; 0.993)
5	0.954	(0.918; 0.992)	0.928	(0.891; 0.966)
10	0.910	(0.842; 0.983)	0.860	(0.793; 0.933)
*Cumulative precipitation (mm)*				
	RR *	(95% CI)	RR *	(95% CI)
1	1.001	(1.000; 1.002)	1.032	(1.019; 1.046)
10	1.010	(1.001; 1.020)	1.172	(1.097; 1.251)
50	1.052	(1.001; 1.105)	1.373	(1.204; 1.565)

* RR were calculated for increase in 1, 5, and 10 °C of dew point and 1, 10, and 50 mm increase of cumulative precipitation.
